# Enhancement in Motor Learning through Genetic Manipulation of the Lynx1 Gene

**DOI:** 10.1371/journal.pone.0043302

**Published:** 2012-11-05

**Authors:** Julie M. Miwa, Andreas Walz

**Affiliations:** 1 California Institute of Technology, Pasadena, California, United States of America; 2 Ophidion, Inc., Pasadena, California, United States of America; University of Chicago, United States of america

## Abstract

The cholinergic system is a neuromodulatory neurotransmitter system involved in a variety of brain processes, including learning and memory, attention, and motor processes, among others. The influence of nicotinic acetylcholine receptors of the cholinergic system are moderated by lynx proteins, which are GPI-anchored membrane proteins forming tight associations with nicotinic receptors. Previous studies indicate lynx1 inhibits nicotinic receptor function and limits neuronal plasticity. We sought to investigate the mechanism of action of lynx1 on nicotinic receptor function, through the generation of lynx mouse models, expressing a soluble version of lynx and comparing results to the full length overexpression. Using rotarod as a test for motor learning, we found that expressing a secreted variant of lynx leads to motor learning enhancements whereas overexpression of full-length lynx had no effect. Further, adult lynx1KO mice demonstrated comparable motor learning enhancements as the soluble transgenic lines, whereas previously, aged lynx1KO mice showed performance augmentation only with nicotine treatment. From this we conclude the motor learning is more sensitive to loss of lynx function, and that the GPI anchor plays a role in the normal function of the lynx protein. In addition, our data suggests that the lynx gene plays a modulatory role in the brain during aging, and that a soluble version of lynx has potential as a tool for adjusting cholinergic-dependent plasticity and learning mechanisms in the brain.

## Introduction

The cholinergic system is a critical modulatory system governing complex processes in the brain. Nicotinic receptors of the cholinergic system bind to the endogenous neurotransmitter acetylcholine, as well as the exogenous drug, nicotine. Activation of such receptors can augment neurotransmitter release, synaptic transmission and enhance synaptic plasticity [1]. Nicotinic receptor activation can also influence some forms of learning and memory, including fear conditioning [2], [3], avoidance learning [4], water maze [5], [6], and motor learning [7], [8]. Several mechanisms exist to control the activity of the cholinergic system [9], [10], [11], [12], [13]. Regulating the activity levels of the cholinergic system, or achieving optimal cholinergic tone in the brain, can be an effective means of controlling the extent of plasticity mechanisms.

One mechanism of cholinergic regulation previously reported is achieved through the modulator, lynx1 [14]. Our studies indicate that lynx1 can form stable complexes with nicotinic receptors, resulting in lower agonist affinity, faster desensitization and slower recovery from desensitization of α4β2 nicotinic receptors, acting as a molecular brake on nicotinic receptor function [15]. Removal of this brake, such as in lynx1 null mutant (lynx1KO) can exhibit features of enhanced cholinergic tone – greater agonist sensitivity and intracellular calcium levels and reduced desensitization in response to nicotine in the brain [9], [16]. The resulting nicotinic receptor hypersensitivity can lead to enhancements in synaptic plasticity [17] and improved fear conditioning [16]. While aged lynx1KO mice were not affected in rotarod performance, there was a significant increase in lynx1KO mice in motor learning when treated with nicotine. These data support the hypothesis that lynx1KO mice are more sensitive to the effects of nicotine than wild-type mice and that this enhanced cholinergic tone resulted in improved learning on the rotarod task. Although lynx1 is widely expressed throughout the brain, it shows particularly high levels in select cells within the cerebellum. Therefore, we sought to investigate further the mechanism of action of lynx1 on nicotinic receptor function, extending some of the initial observations of enhanced motor learning into the cerebellum by manipulating the levels of the lynx1 gene.

The rotarod task measures motor coordination and can also measure motor learning [18]. The cerebellum is highly implicated in the functioning of this task, and it is a well documented site of action for other learning paradigms [19] such as conditioned eye-blink [20], [21], [22]. The main function of the cerebellar circuit is to refine sensory and motor information – integrating these inputs to fine tune motor activity to aid in motor coordination [23]. The Purkinje cell is the main output neuron in the cerebellar cortex and sends inhibitory signals to the deep nuclear neurons of the cerebellum. The cerebellar Purkinje cell is a highly integrative cell, which segregates its many afferent inputs into discrete subdomains. Its main excitatory inputs are received by parallel fibers synapsing onto distal dendrites of Purkinje cells, and climbing fibers synapsing onto the somatodendritic region near to Purkinje cell bodies. The majority of inhibitory inputs onto Purkinje cells arise from stellate and basket cells in the molecular layer [19], synapsing onto Purkinje proximal dendrites and cell bodies, respectively. Synaptic alterations during the pairing of presynaptic stimuli can be induced by removing inhibition, indicating that alterations in excitatory/inhibitory balance could influence synaptic plasticity in this circuit. Therefore, the cerebellar cortex is a useful region to probe the effect of cholinergic modulation of neuronal circuits.

The cholinergic system has been reported to mediate release of neurotransmitter in the cerebellum [24], [25], [26], [27], [28], and therefore influence cerebellar activity [29], [30] in normal [31] and abnormal [32] brain function. Both muscarinic and nicotinic receptors have been localized in the cerebellar cortex [33]. In the rat cerebellum, α4 nicotinic receptor subunit immunoreactivity has been identified in the cell bodies in the molecular, granule and Purkinje cell layers and in pre-synaptic terminals to Purkinje cells [34]. α7 nicotinic receptor subunit immunoreactivity can be found in rat Purkinje cell and granule cell dendrites, but not in granule cell somata [35]. Several different subtypes of nicotinic receptor have been reported in the cerebellum, approximately half of which are α4β2* receptors (composed of α4β2, α3α4β2, and α4β2β4), and approximately half of which are α3β4* receptors (composed of α3β4, α3β2β4 α3β2β4, and α3α4β4 [36]. Because of this differential distribution, selective modulation of these subtypes would likely have a complex effect on the function of the cerebellar circuit. In the cerebellum, lynx1 message has been found at high levels in deep nuclei, and at moderate levels in Purkinje cells. Lynx1 protein in the cerebellar cortex is restricted to the somatodendritic compartment of Purkinje cells, a neuronal subdomain that is correlated with a defined set of afferent inputs, climbing fiber excitatory input, and stellate/basket neuron inhibitory input.

Lynx genes and family members consist of both secreted [37], [38], [39], [40], [41], [42], and membrane-bound variants [9], [43], [44], [45], [46], [47], [48], [49], [50]. The majority of the mammalian family members are membrane-bound peripheral membrane proteins, anchored to the membrane through sugar-lipid interactions via a glycosylphospholipid (GPI)–linked anchor. Previously we reported that purified lynx1 protein has modulatory capability over nicotinic receptors in vitro [14], distinct from effects of co-expression of the full-length membrane anchored form of the protein [15]. To further probe the biophysical mechanism of action of the membrane anchor for lynx function, we employed genetic engineering strategies to express membrane bound and secreted lynx isoforms in the brains of mice utilizing three genetically modified mouse lines.

## Results

**Figure 1:**
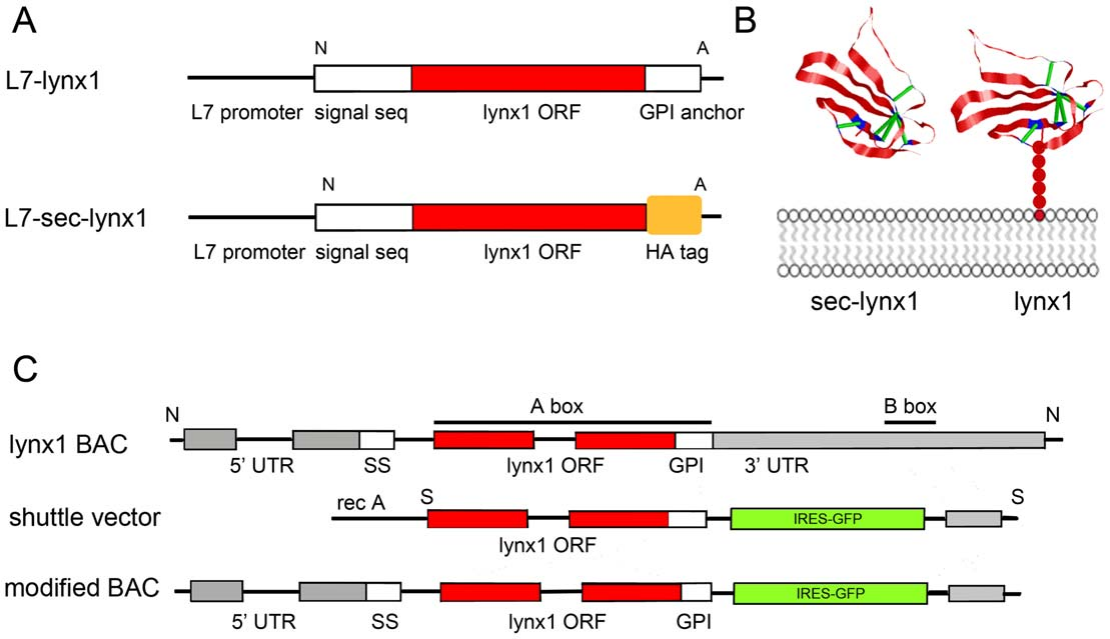
Summary of three transgenic lynx1 constructs: L7 lynx1, L7 secreted lynx1, and lynx1 BAC. (A) L7-lynx1 construct, upper, utilizing a pcp (L7) promoter sequence in front of a full length lynx1 cDNA contained the full length lynx1 coding sequence. Lower, L7-sec-lynx1, pcp (L7) promoter driving expression of a secreted variant of lynx1, lacking the final asparagine residue that makes up the mature form of lynx1, and lacking the GPI-anchor hydrophobic consensus sequence. An in-frame HA sequence replaces the GPI anchor sequence. (B) Representative structural model of lynx1 (right hand molecule) with associated GPI-linked tether (red circles) to the plasma membrane (grey). Model is based on the NMR structure of lynx1 [56]. Left hand molecule, the secreted variant of lynx1 can translocate freely across the plasma membrane and diffuse into the extracellular and/or synaptic space. (C) BAC modification strategy for the generation of a lynx1 modified BAC transgenic mouse line.

A secreted version of the lynx1 protein lacking the consensus sequence for the GPI attachment (sec-lynx1, Figure 1A) was used to construct an over-expression transgenic line. In the native lynx1 gene, a leading signal sequence directs the polypeptide across the membrane, while the C-terminal hydrophobic consensus sequence directs attachment of the nascent polypeptide to the GPI anchor. During the process of biosynthesis of GPI-anchored proteins, the C-terminal signal sequence directs the nascent polypeptide to the membrane until the attachment of the anchor occurs. Upon attachment of the GPI moiety, the embedded GPI consensus sequence is cleaved from the mature polypeptide. By removing the consensus sequence for the attachment of the GPI-moiety, the lynx polypeptide variant would be directed across the plasma membrane, but not anchored there.

**Figure 2:**
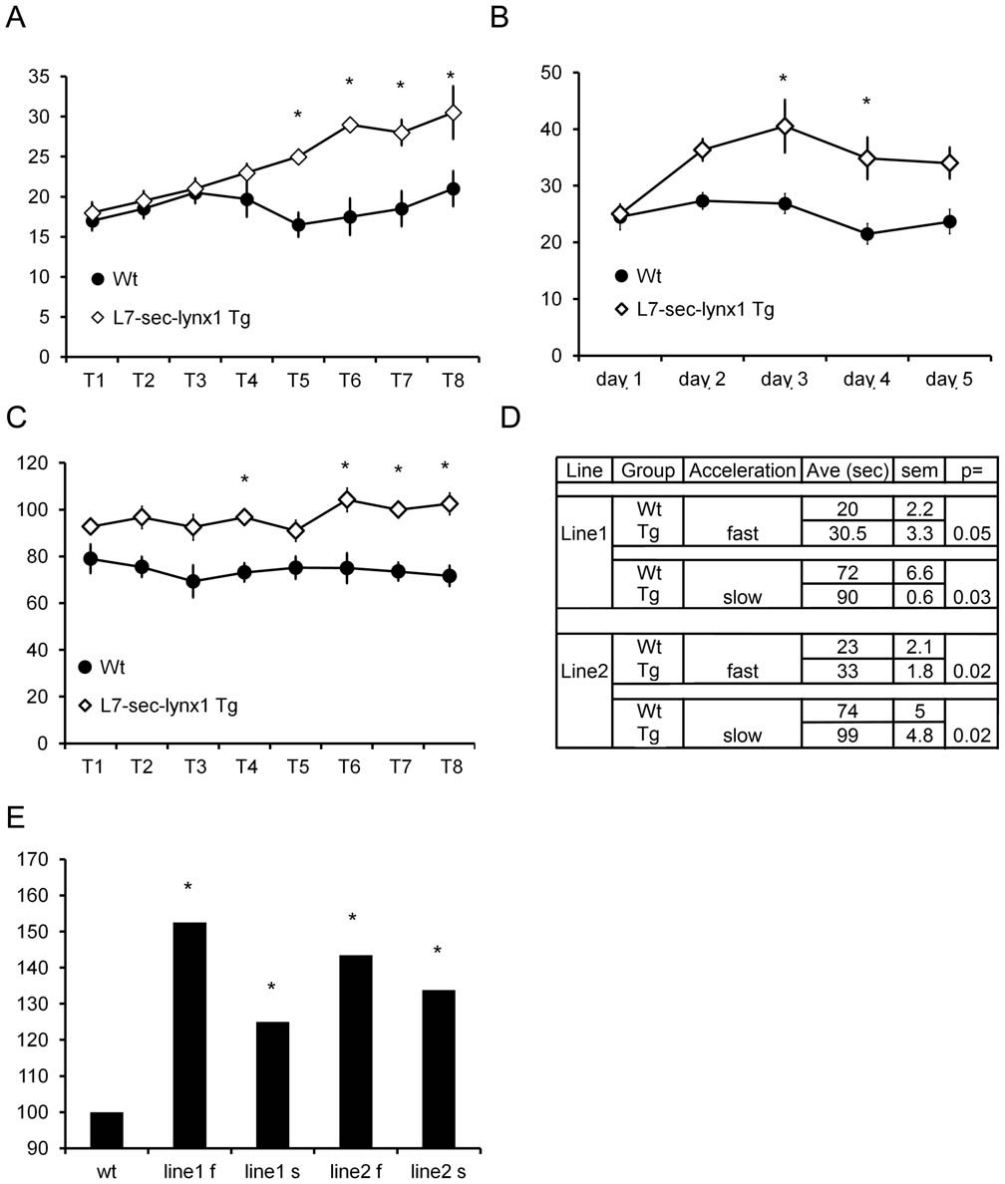
Rotarod performance for L7-sec-lynx1 transgenic mice. (A) Line 1. No differences in performance were observed initially, but the transgenic mice (L7-sec-lynx1 Tg) outperformed their wild-type littermates beginning at the 5^th^ trial. These data indicate that there are no motor performance differences in the transgenic mice vs. wildtype mice, but that there is a significant improvement in motor learning behavior. Y axis is in seconds; X axis is trial number. P<0.05. (B) Line 1. Motor performance over five consecutive training days. Average of all trials per day indicates a significant performance enhancement in L7-sec-lynx1 Tg as compared to their wild-type littermate controls. Y axis is in seconds, X axis is in days. (C) Line 2. Motor performance using a slow acceleration rotarod paradigm (0.1 RPM/sec). Significant enhancements in rotarod performance in L7-sec-lynx1 Tg mice were observed in latter trials. Y axis is in seconds; X axis is in trials. P<0.05. (D) Table of data collected for two independent founder lines of the L7-sec-lynx1 transgene tested on an accelerating rotarod paradigm, with two separate acceleration paradigms of the rotarod test (modes) used, slower (0.1 RPM/sec.) and faster (1 RPM/sec.) accelerating paradigm. In both paradigms, both L7-lynx1 transgenic mouse lines demonstrated a significant enhancement in motor learning but no differences in baseline motor performance. (E) Summary of the effects of motor learning on the two lines of the same L7-sec-lynx1 transgenic mice. The maximal daily performance from all training days is plotted as a percentage relative to wild-type. “f” and “s” suffixes refer to the faster or slower acceleration, respectively.

To assess the consequence of an alternate isoform to the functioning of neuronal circuits, we used the cerebellar Purkinje cell specific promoter, L7, to drive expression of this secreted lynx variant (L7-sec-lynx1) (Figure 1A, lower). We then employed a learning assay that is dependent on the action of the Purkinje cell, the accelerating rotarod paradigm, a test of motor coordination and learning. Two independent founder lines were bred to C57BL/6 mice, and the progeny were tested for motor coordination and learning. Founder line one of the L7-sec-lynx1 transgenic mouse line demonstrated no basal differences in motor performance in an initial trial as compared to wild-type littermate control mice. Upon subsequent training trials, however, L7-sec-lynx1 mice showed a significant improvement by the fifth trial on the first day of training (Figure 2A). On subsequent training days, the mutant mice maintained the improved rotarod ability across five days of training (Figure 2B). To determine whether motor alterations are due to learning enhancements vs. differences in motor function or coordination, two different learning parameters were used, a slow and a fast acceleration speed (Figure 2C). In both conditions, progeny from two independent transgenic founder lines of the same transgene demonstrated a learning enhancement after training (Figure 2D–E). Because improvements in rotarod performance were not observed until latter trials, these data are indicative of improved motor learning, as opposed to motor coordination or performance. Motor learning enhancements were observed in a second independent founder line for the L7-sec-lynx1 transgene, supporting a specific effect of the soluble lynx polypeptides, as opposed to differential integration sites of the lynx transgenic lines (Figure 1D).

**Figure 3:**
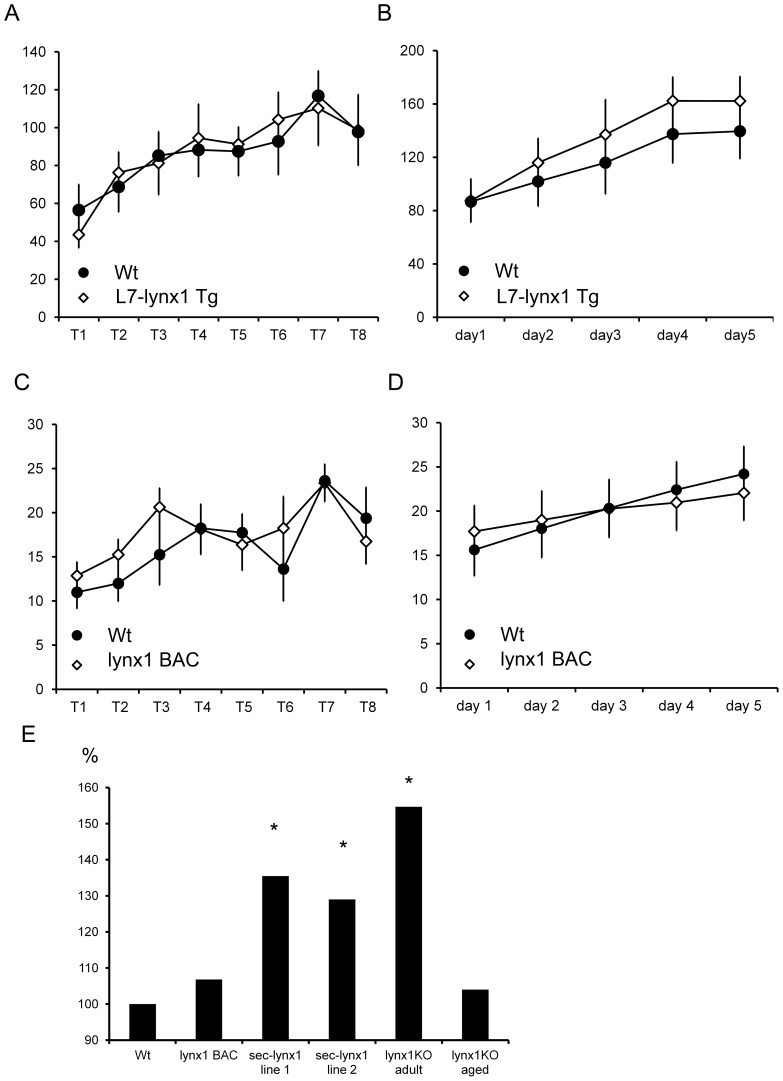
Comparison of motor learning effects across several genetically modified lynx lines. Overexpression lines, L7-lynx1 and lynx1BAC mice, which over-expressed full length lynx1 in Purkinje cells, and lynx1-expressing cells, respectively, did not display significant differences in motor learning. (A) L7-lynx1 transgenic mice, overexpressing full length lynx1 in Purkinje cells, showed no differences in motor performance or learning as compared to their wild-type counterparts. Y axis is in seconds, X is in trials. (B) L7-lynx1 transgenic mice exhibited no learning improvements over that of wild-type littermates. Y axis is in seconds, X axis is in days. (C) lynx1 BAC transgenic mice showed no difference in motor performance as compared to wild-type littermates in initial training trials on the rotarod. Y axis is in seconds, X is in trials. No significant differences were observed. (D) On subsequent training days, lynx1BAC mice did not show a difference in motor learning. Y axis is in seconds. No significant differences were observed. (E) Comparisons of motor learning across lines. Data is represented for each line as a percentage over the performance of their wild-type counterparts. The improvement in motor learning of L7-sec-lynx1 transgenic mice contrast with expression of full-length lynx1 using lynx1BAC transgenesis. Rotarod motor learning was also sensitive to removal of lynx (lynx1 KO), and demonstrated a significant increase over wild-type controls. Aged mice, (>1 yr), showed no motor enhancements unless treated with nicotine [16]. Y axis is percentage over wild-type controls. Data depicted represent the seventh training trial for each group.

A second transgenic construct was generated utilizing the same L7 promoter to over-express the full-length version of the lynx1 polypeptide, L7-lynx1 transgenic mice (Figure 1A, upper). When expressed in transgenic mice, the full-length normal variant of lynx1 would attach at the plasma membrane through its GPI anchor, only in cerebellar Purkinje neurons. As with the L7-sec-lynx1 transgenic mice, the L7-lynx1 transgenic mice demonstrated no basal differences in motor performance, on the first trial. In contrast, however, motor performance in the L7-lynx1 transgenic mice never demonstrated significant differences in motor learning or performance across any of the training days (Figure 3A and B). These data indicate that lynx1 protein levels in Purkinje cells of wild-type are not limiting, as increasing lynx1 in this cell type has no effect on this behavior.

In a third independent transgene, we employed a bacterial artificial chromosome (BAC) recombineering strategy using the lynx1 genomic locus to over-express full-length lynx1 under control of the lynx1 promoter. When expressed in the BAC transgenic mice, lynx1BAC transgenic mice, full-length membrane bound lynx1 would be expressed in all lynx expressing cells of mice. These lynx1BAC transgenic mice were tested in the same rotarod paradigm, and showed no differences compared to wild-type mice on the initial test day, indicating no significant differences in motor performance (Figure 3C). On subsequent training days, lynx1–BAC transgenic mice displayed similar abilities as compared to their wild-type counterparts, indicating lynx1 BAC mice were not improved in motor learning (Figure 3D). Results from these two overexpression transgenic lines indicate that levels of the lynx1 protein are not limiting for the full expression of the behavioral phenotype.

We previously observed that motor learning was enhanced when aged lynx1KO mice were treated with a chronic course of nicotine (Miwa et al., 2006). In comparing naïve adult mice (8–12 weeks of age) with aged mice (>9 months), we found a greater enhancement of motor learning in young adult mice, as compared to aged lynx1KO mice (Figure 3E). These data indicate that manipulations to inhibit lynx dosage may be more advantageous than addition of lynx levels, and that there is an age related effect of lynx1. Alterations in anxiety cannot account for the performance differences in lynx1KO mice, as they were normal in light-dark box (data not shown), open field, or elevated-plus maze assays [16]. These data, taken together indicate ectopic expression or expression of lynx variants leads to increases in motor learning.

## Discussion

In this study we probed the ability of a lynx polypeptide to modulate neuronal circuits using conventional and BAC transgenesis. Previous studies show that lynx helps to regulate the level of cholinergic activity through direct interactions with nicotinic receptors. The present data demonstrate that a non-membrane bound form of the lynx1 polypeptide, secreted lynx1, has distinct, positive neuromodulatory effects on motor learning. The motor enhancement due to expression of secreted lynx1 occurs in the context of wild-type levels of the normal protein. Over-expression of full length lynx1 did not produce differences in motor learning as compared to wild-type mice, indicating that lynx levels do not limit the expression of this behavior. Furthermore, differential effects of full length vs. secreted lynx1 overexpression in cerebellar Purkinje cells suggest that the GPI-anchor is a critical component of normal lynx1 function. Finally, because lynx1KO mice display similar motor learning enhancements as the sec-lynx1 Tg lines, this supports the idea that the secreted version of lynx1 act differently from the full length, membrane-bound version. We hypothesize that secreted lynx1 can act as a dominant negative and compete off wild-type membrane-bound lynx1, creating an effect similar to the lynx1KO mice. Alternatively, the secreted version could reach new sites in the cerebellum not normally reached by the lynx1 protein. Taken together, these data suggest that delivery of a lynx polypeptide could therapeutically regulate cholinergic activity to achieve enhancements in synaptic plasticity, learning and/or memory.

Because overexpression of lynx had no effect on either L7-lynx1 or lynx1BAC transgenic mice, we can conclude that lynx1 levels are not limiting in Purkinje cells with respect to rotarod learning ability. Rather, we have seen that the brain is more sensitive to loss of lynx function rather than overexpression. Improved rotarod performance can also be elicited in adult lynx1KO, a sign of enhanced motor learning ability. We can partially rule out other factors, such as anxiety, that could contribute at a motivational level to the performance of these mice. We cannot completely exclude the possibility that fatigue could differentially influence motor performance between the two genotypes; the improved ability in the L7-sec-lynx mice –expressed only in Purkinje cells- indicates that the site of change is within the cerebellum, decreasing the likelihood that muscle fatigue is a factor in our results. Furthermore, we have not discerned a notable drop-off in performance from the first training trial to the last of each day, potentially an indicator of muscle fatigue, between the different genotypes. Lastly, adult lynx1KO mice demonstrated motor learning enhancements, as compared to aged lynx1KO mice, which showed performance augmentation only with nicotine treatment [16]. Given the significant degeneration in lynx1KO over one year of age, it is likely that motor loss of function accompanies the degeneration in aged lynx1KO.

**Figure 4:**
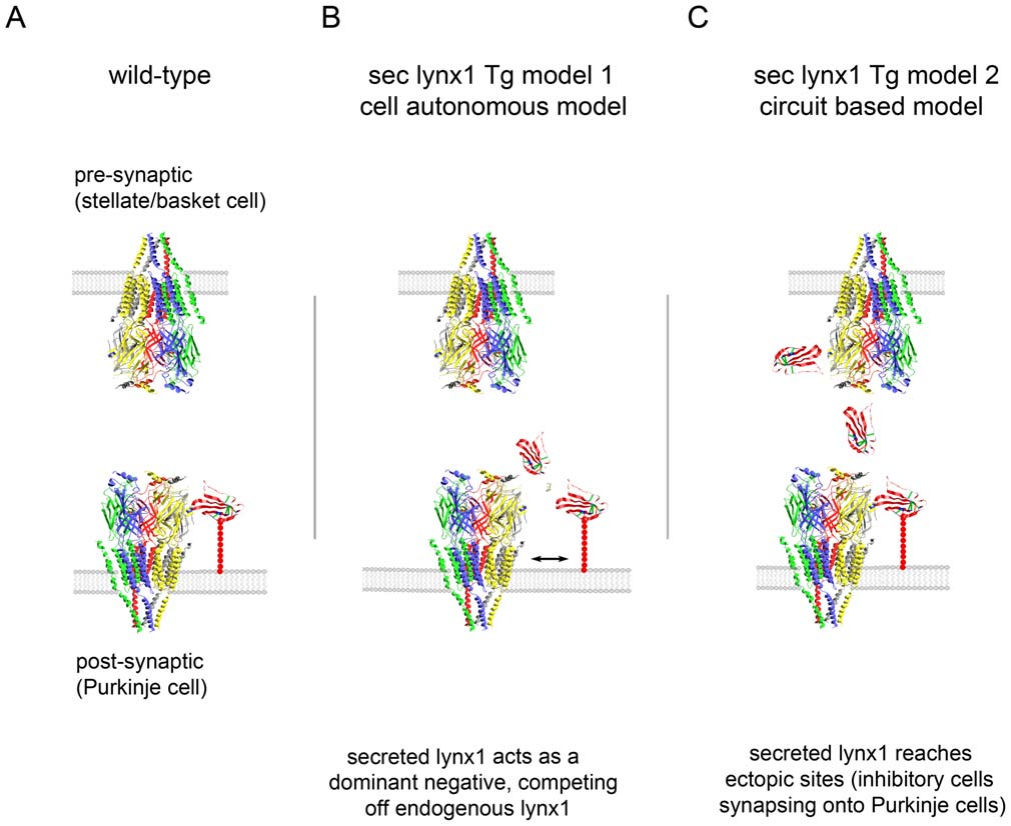
Two schemes for possible modes of action of secreted lynx on nicotinic receptors within synapses. (A) Schematic diagram of a synapse in WT mice. Schematic model of nicotinic receptors and lynx1 interaction at a Purkinje cell synapse. Models are based on the crystal structure of AChBP [57] and the NMR structure of lynx1 [56]. Lynx1 is depicted as binding at the subunit interface of the pentameric channel, based on α-bungarotoxin binding. Lynx1 expression is expressed in the post-synaptic cell (Purkinje cell), and is not expressed in the presynaptic neuron (stellate/basket neuron). Tethered to the membrane by it GPI-anchor, lynx is depicted as having access to a nicotinic receptor binding site at the post-synaptic face only. (B) Schematic model 1 of sec-lynx1 function – cell autonomous, dominant negative model. Schematic representation of Purkinje cell synapses in L7-sec-lynx1 Tg mice. Binding of secreted lynx1 to the same subunit interface of the nicotinic receptor could compete off the binding of native full-length GPI-anchored lynx1, and thereby exert a dominant negative effect. This model implies that the secreted version of lynx1 has either no effect or a differential function as compared to the membrane bound version of lynx1, but maintains nicotinic receptor binding capability. (C) Schematic model 2 of sec-lynx1 function – circuit based, ectopic expression model. In this model, the soluble lynx1 secreted from the post-synaptic Purkinje cells diffuses extracellularly, accessing nicotinic receptors located on terminals of pre-synaptic neurons (stellate/basket cell). In this model, the ectopic expression of lynx1 in pre-synaptic sites can lead to suppression of activity or neurotransmitter release, leading to dis-inhibition onto Purkinje cells. This dis-inhibition can lead to alterations in excitatory/inhibitory balance and motor learning.

The cerebellum has been implicated in some forms of plasticity and learning. Purkinje cell firing is controlled through a balance of excitatory vs. inhibitory inputs [19]. Synaptic alterations during the pairing of presynaptic stimuli can be induced by removing inhibition, indicating that modulating inhibition could influence synaptic plasticity in this circuit. We have considered two potential models of action of secreted lynx in the L7-sec-lynx1 transgenic mice (Figure 4), which can be broadly categorized as a cell autonomous model (Figure 4B), and a circuit based model (Figure 4C).

Cell autonomous, dominant negative model: In normal cases, lynx1 is bound to the cell surface of Purkinje cells, and is not expressed in presynaptic neurons (Figure 4A). In the cell autonomous model (Figure 4B), the secreted lynx1 could outcompete the binding of the GPI-tethered lynx1 to the nicotinic receptor in the Purkinje cell, creating a dominant negative effect and leading to nicotinic receptor hypersensitivity. The similarity in learning enhancements between the secreted Tg and the lynx1KO support this model. This competition could occur on the cell surface, or during the trafficking of the receptor. In previously published reports, however, secreted lynx amplified ACh evoked current in α4β2 nicotinic receptor-injected oocytes, when no lynx was present, indicating that at least part of the function of lynx has the potential to function through direct action on receptor gating, and not only through a dominant negative competition of endogenous lynx1.

Circuit based alteration, ectopic expression model (Figure 4C): Because the secreted variant of lynx1 is not anchored to the plasma membrane, it has the potential to be released into the synaptic or extracellular spaces and reach sites otherwise not available to the native, GPI-bound version of wild-type lynx1. We have detected lynx1 binding sites in the cerebellum that do not correspond to lynx expression. Purified lynx1-fc binds to sites in the cerebellar molecular layer (i.e. inhibitory stellate and basket neurons), while lynx1 gene expression is confined to Purkinje cells in the cerebellar cortex. The untethered lynx1 variant in the L7-sec-lynx1 transgenic line could reach stellate/basket neurons ectopically. We would expect this to influence neurotransmitter release from terminals of stellate/basket neurons, due to the modulatory properties of lynx. Reducing nicotinic receptor function on stellate and basket cell interneurons could result in a disinhibition of Purkinje cells. It has been suggested that synaptic inhibition onto Purkinje cells can fine-tune cerebellar circuit activity [51], mediate synaptic plasticity [52] and enhance motor learning functions [53]. Alterations in the firing patterns of Purkinje cells will have consequences to motor coordination and learning. Purkinje cells are the major output of the cerebellar cortex, and loss of Purkinje cells activity through dysfunction can lead to a reduction of motor coordination and learning [54]. Manipulation of the lynx gene has the potential to alter the excitatory/inhibitory balance within the cerebellar cortex. Therefore, reduced inhibitory output onto Purkinje cells, could create a permissive environment for synaptic alterations underlying motor learning events.

The experiments conducted here demonstrate that membrane tethering is important for the proper function of lynx1. GPI-anchored proteins have an affinity for a specialized lipid domain referred to as lipid rafts, and as such undergo differences in sorting than transmembrane proteins [49]. It has been proposed that GPI-anchored proteins are capable of directing receptors they bind to into rafts, which can influence sorting, nucleation sites of receptor complexes, and more [55]. Because of the possibility of specific sorting the GPI anchor can confer, the secreted lynx variant in the L7-sec-lynx1 transgenic mouse line could fail to elicit the normal function of membrane-bound lynx1 due to incorrect localization and/or number of receptors. In vitro studies on the action of lynx1 on α4β2 nicotinic receptors indicate that application of purified soluble form of lynx has different effects in oocytes as compared to full-length lynx1 when co-expressed with α4β2 nicotinic receptors [14], [15], but whether these differences could be due to receptor sorting differences remains to be determined.

In this particular study, we employed the rotarod assay test of motor performance and learning, but our analysis with the lynx1 knock-out mouse line suggests that lynx1 is important in other learning and memory functions [16] and plasticity mechanisms as well [17]. Therefore, a therapeutic lynx polypeptide may be useful in treating memory dysfunctions across multiple modalities.

## Methods

### Transgenic constructs

#### L7-sec-lynx1 transgenic construct

A non-membrane bound version of lynx1 was subcloned behind the pcp2 (L7) promoter to drive specific expression of a secreted form of lynx1 specifically in Purkinje cells. A Not1-Apa1 fragment from a lynx1 cDNA construct, CMV2611, was subcloned into the pCEVII expression vector. The template from which the Not1-Apa1 fragment was digested, CMV2611, was constructed through PCR amplification of a 4.5 kb lynx1-containing cDNA, GC26.2 with Not1 and Apa1 restriction sites designed in the 5′ and 3′ primers, respectively. The 3′ primer produced an in-frame HA tag and stop codon after the last cysteine residue that makes up the ly-6 consensus motif, deleting the final asparagine residue before the consensus sequence for the attachment of the GPI-anchor. DNA was linearized with Apa1, and the fragments were purified by cesium chloride centrifugation and phenol chloroform extraction. The construct was introduced into CBA/C57BL/6 hybrid mice by DNA injection into embryos using standard transgenic techniques.

#### L7- lynx1 transgenic construct

Overexpression of lynx1 in Purkinje cells was generated by subcloning a lynx cDNA fragment from a full-length lynx1 construct, CMV-269. A Not1-Apa CMV-269 fragment was subcloned into pCEVII and purified as above. The CMV-269 construct was generated using the same 5′ primer as for CMV-2611, and a 3′ primer pair at a similar position on the lynx1 gene, near or at the start of the 3′UTR.

#### Lynx1BAC transgenic construct

A lynx1 open reading frame probe was used to screen a mouse BAC library for a BAC clone containing the lynx1 gene. (BAC clone 77K17). The PCR primer pair used to generate CMV269, was used to amplify a genomic fragment of the lynx BAC clone 77K17 to create a 5′ homology arm (A box) flanked by XhoI sites, and subcloned with Xho1 into an IRES-GFP building vector (pBV1). The B box was created by subcloning a Xba1 fragment from lynx1 cDNA M25 in exon IV into the pBV1. The entire building vector insert was cloned as a Sal1 fragment into the recA shuttle vector (pSV1recA). The pSV1recA vector containing the lynx1 homology arms was transformed into 77K17 BAC-containing bacterial cells and plated on tetracycline and chloramphenicol plates at 30°C to select for co-integration of the psV1recA vector. To ensure complete homologous recombination, plates were incubated at 43°C to remove the recombination capacity of the cells, and to thus remove incompletely integrated shuttle vectors. Correct cointegration was verified by Southern blot analysis. Co-integrates were then plated on chloramphenicol plates at 43°C to induce resolution, and chloramphenicol and fusaric acid at 37°C to select for loss of the tet gene. Resolved BACs were screened by Southern blot using radiolabeled probes of each of the lynx1 open reading frame, A box sequence, and B box sequences. DNA preparation was conducted in the same manner as the L7-sec-lynx1 construct.

### Mouse Breeding

Progeny of the positive transgenic founder mice were bred with BL/6 mice to produce F1 positive and wild-type littermates, and maintained under a standard 12 hour light-dark cycle. Positive F1 transgenic mice and their wild-type littermates were tested on rotarod at 7–12 weeks of age. Testing was conducted on age and sex matched littermate controls. A fourth transgenic mouse line was generated, a secreted form of lynx driven by the lynx1 genomic regions contained on BAC 77K17, but the founder mice did not transmit the transgenic allele to their progeny and were not tested (data not shown). Lynx1KO mice were tested at 8–12 weeks of age (adult mice), and aged mice were tested over 12 months of age. Rockefeller IACUC committee reviewed the procedures used in these experiments and approved this study. The animals were handled according to Animal Welfare guidelines, and were not subjected to any procedures that were painful or induced suffering.

### Rotarod motor learning paradigm

Genetically modified animals were tested for performance in an accelerating rotarod test. In the rotarod assay, mice are placed on a rotating rod at the slowest speed, at 0.1 RPM, and accelerated at a constant rate of acceleration, either 1.0 RPM/sec. rate of acceleration (fast acceleration) or 0.1 RPM/sec. (slow acceleration). The latency of each trial was measured as the time the mouse stayed on the rod until it either fell off of the rotating rod, or clung to the rod without running for an entire revolution. Eight trials per day were tested for five days. Initial trials of testing evaluate motor coordination in the mice, whereas subsequent training trials measure the adaptability of the circuit, and thus test motor learning or plasticity. Similar results were obtained with four trials per day on the first training day.
